# The prognostic value of tumor necrosis in patients undergoing stereotactic radiosurgery of brain metastases

**DOI:** 10.1186/1748-717X-8-162

**Published:** 2013-07-03

**Authors:** Kristina Martens, Thekla Meyners, Dirk Rades, Volker Tronnier, Matteo Mario Bonsanto, Dirk Petersen, Jürgen Dunst, Kathrin Dellas

**Affiliations:** 1 Department of Radiotherapy, University of Luebeck, Ratzeburger Allee 160, Luebeck 23538, Germany; 2 Department of Neurosurgery, University of Luebeck, Luebeck Germany; 3 Institute of Neuroradiology, University of Luebeck, Luebeck Germany; 4 Department of Radiotherapy, University of Kiel, Kiel, Germany; 5 Department of Radiotherapy, University of Copenhagen, Copenhagen, Denmark

**Keywords:** Radiotherapy, Stereotactic radiotherapy, Brain metastasis, Prognostic factor, Necrosis

## Abstract

**Background:**

This retrospective study investigated the outcome of patients with brain metastases after radiosurgery with special emphasis on prognostic impact of visible intratumoral necrosis on survival and local control.

**Methods:**

From 1998 through 2008, 149 patients with brain metastases from solid tumors were treated with stereotactic radiotherapy at Luebeck University. Median age was 58.4 years with 11%, 78%, 10% in recursive partitioning analysis (RPA) classes I, II, III, respectively. 70% had 1 metastasis, 29% 2-3 metastases, 2 patients more than 3 metastases, 71% active extracranial disease. Median volume of metastatic lesions was 4.7 cm^3^, median radiosurgery dose 22 Gy (single fraction). 71% of patients received additional whole-brain irradiation (WBI). All patients were analyzed regarding survival, local, distant failure and prognostic factors, side effects and changes in neurologic symptoms after radiotherapy. The type of contrast-enhancement in MR imaging was also analyzed; metastatic lesions were classified as containing necrosis if they appeared as ring-enhancing with central areas of no or minimal contrast enhancement.

**Results:**

Median survival was 7.0 months with 1-year and 5-year survival rates of 33% and 0.4%, respectively. Tumor necrosis (ring-enhancement) was visible on pretreatment MRI scans in 56% of all lesions and lesions with necrosis were larger than non-necrotic lesions (6.7 cm^3^ vs. 3.2 cm^3^, p = 0.01). Patients with tumor necrosis had a median survival of 5.4 months, patients without tumor necrosis 7.2 months. Local control rate in the irradiated volume was 70%, median survival without local failure 17.8 months. Control in the brain outside the irradiated volume was 60%, median survival without distant failure 14.0 months. Significant prognostic factors for overall survival were KPS (p = 0.001), presence of tumor necrosis on pretreatment MRI (p = 0.001) with RPA-class and WBI reaching marginal significance (each p = 0.05). Prognostic impact of tumor necrosis remained significant if only smaller tumors with a volume below 3.5 cm^3^ (p = 0.03). Side effects were rare, only one patient suffered from serious acute side effects.

**Conclusions:**

Results of this retrospective study support that stereotactic radiotherapy is an effective treatment option for patients with metastatic brain lesions. The prognostic impact of visible tumor necrosis (ring-enhancement) on pretreatment MRI scans should be further investigated.

## Background

In the last decades, stereotactic radiosurgery (SRS) has become a standard treatment procedure for the management of certain intracranial lesions, such as brain metastases, and malignant as well as benign brain tumors [1-12]. Originally developed by Lars Leksell in 1951 as a substitute for direct surgical intervention, radiosurgery is a technique that involves single treatment radiotherapy precisely focused at intracranial targets. The precision of stereotactic positioning combined with the steep dose gradients allows sparing normal tissue while reliably destroying tissue within the target volume. To apply stereotactic radiotherapy, either a dedicated system (e.g. Gamma Knife or CyberKnife) or a modified linear accelerator [13] is used. The procedure is more time-consuming as compared to a standard radiotherapy fraction [14]; however, treatment is convenient for patients, and many of them prefer the use of stereotactic radiotherapy instead of surgery because of lower morbidity and side effects but similar rates of tumor control [15-22].

A recent meta-analysis has evaluated the outcome after SRS in a variety of neurooncological indications including vestibular schwannoma (37 studies with a total of 3677 patients), meningioma (15 studies with a total of 2734 patients), metastatic disease (27 studies with a total of 2679 patients), and glioblastoma (11 studies). This analysis found an overall survival in patients with metastatic brain lesions of 5-14 months from time of SRS and a 1-year overall survival rate ranging from 15-55% with a local control rate of 59% to 97% [23].

In a subset of metastatic lesions, spontaneous necrosis is visible on magnetic resonance imaging (MRI) [4]. The presence of visible necrotic areas seems to be a poor prognostic factor in a variety of cancer sites and brain tumors, especially in malignant gliomas [6]. However, little is known about the impact of necrosis in metastases which are treated with radiosurgery. This study investigates a possible association between tumor necrosis and survival or tumor control after stereotactic radiosurgery. Furthermore, this study was designed to review the effectiveness and to determine prognostic factors for survival and local control as well as the rate of side effects of stereotactic radiotherapy in patients with brain metastases treated over a period of more than ten years at the Luebeck University.

## Methods

The retrospective study was conducted according to the principles of the Declaration of Helsinki and to good clinical guidelines. The study has been notified by the Ethics Committee, University of Luebeck.

### Patient population

From 1998 through 2008, 273 patients received radiosurgery or fractionated stereotactic radiotherapy in the Department of Radiotherapy at the University of Luebeck. 103 patients had benign brain tumors (mainly vestibular schwannoma) and 21 patients were treated because of malignant primary brain tumors (mainly recurrent glioblastoma) or lesions of the base of the skull. The remaining 149 patients were treated for brain metastases and are the basis of this analysis. Table 1 shows the distribution of primary tumors. A detailed description of the patient’s characteristics is listed in Table 2.

**Table 1:** Distribution of primary tumors

**Type of tumor**	**N (%)**
**Brain metastases**	**149**
Lung cancer	77
Breast cancer	22
Melanoma	16
Renal cancer	12
Colon cancer	11
Ovarian cancer and uterine cancer	4
CUP	3
Others (seminoma, prostate-cancer, follicular thyroid cancer, AML)	4

**Table 2:** Characteristics of patients

**Parameter**	**N**	**(%)**
Age, years		
Median age	58.4	
Range	23-83	
Status of primary lesions		
Active	106	(71)
Controlled	43	(29)
RPA-class		
I	17	(11)
II	117	(78)
III	15	(10)
KPS		
Median		(80)
≥ 90%	88	(59)
< 90%	61	(41)
Number of lesions		
1	103	(70)
2-3	43	(29)
4-6	2	(1)
Median dose	22.2	Gy
Median tumor size	4.7	cm^3^
Range	0.01-33	cm^3^
Treated with WBI	78	(52)
Therapy of primary lesion		
Chemotherapy	117	(79)
Radiotherapy	60	(40)
Operation	96	(64)
Localization of lesion		
cerebellum	15	(10)
diencephalon	5	(3)
brain stem	3	(2)
frontal	47	(31)
parietal	33	(22)
occipital	16	(11)
temporal	18	(12)
basal ganglia	5	(3)
other localization	7	(5)

Abbreviations:

*KPS* Karnofsky performance status, *RPA* Recursive partitioning analysis, *WBI*, Whole brain irradiation.

### Treatment

All patients were treated with a linear accelerator (Clinac 2100 C, Varian Inc) with 6 MeV photons and a micro-MLC. As planning software, BrainScan (BrainLAB GmbH) was used. In the first years, invasive fixation was routinely used, but since 2005, all patients were treated with individually customized mask systems.

136 patients (91%) were treated with a single fraction with doses between 15 and 27 Gy, the median dose was 22 Gy, prescribed to the surrounding isodose (mostly the 70%-isodose). Patients without whole brain irradiation (WBI) received doses of 20 to 27 Gy, whereas in patients with prior or planned WBI, the dose of the radiosurgical fraction was reduced to 15 to 18 Gy. 13 patients were treated with hypofractioned radiotherapy regimens with three to six fractions and single doses of 8 to 12 Gy per fraction.

### Tumor necrosis

To determine tumor necrosis, T1-weightend contrast-enhanced MR-images were required analyzing existence of visible enhancement. Necrosis was defined as non-perfused areas at central location of the tumor and a ring enhancement. A volume calculation was not performed.

MR examinations were performed by different radiologists (not exclusively neuroradiologists) in different institutions and were not standardized. Therefore, this analysis was not blinded and the classification of necrosis was done on the basis of written records.

### Statistical analysis

Patients were routinely followed in the department, normally in intervals of 6 and 12 months except patients in poor general condition. The median follow-up interval was 12.9 months (range 0.16 - 124 months). Survival data were determined also by the regional cancer registry (Schleswig-Holstein Cancer Registry); however, 22 patients were lost to follow-up. Furthermore, local and distant control of 41 patients could not be determined.

All patients were analyzed regarding survival, local, distant failure and prognostic factors, side effects and changes in neurologic symptoms after radiotherapy. The survival time was calculated from the first day of radiotherapy to the day of the patient’s death or the detection of a recurrence. Concerning failures in the brain, time to local failure (recurrence in the irradiated volume) as well as time to any brain failure elsewhere were determined. The progression of metastases had to be detected by cerebral computertomography (CCT) or MRI. Different prognostic factors and their influence on the clinical endpoint were analyzed, namely age, the localization of the lesion in the brain, the number of brain metastases, the Karnofsky performance status (KPS), the recursive partitioning analysis (RPA) class, tumor necrosis, which was detected by MRI or CCT, the total dose in Gy, gender, improvement of symptoms, the status of the primary (active, if the primary tumor is uncontrolled or other extracranial metastases were detected), tumor size, the number of extracranial organs with metastatic involvement, additional WBI, the histology of the primary lesion and the treatment of the primary tumor (chemotherapy, surgery or radiotherapy). Also, side effects and symptomatic changes after radiotherapy were analyzed. In addition, it was distinguished between improvement, persistence or deterioration of symptoms.

Survival was estimated with the Kaplan-Meier method and log rank tests were used to evaluate the effects of patient characteristics and treatment factors on the clinical endpoints. Univariate and multiple cox proportional hazards analysis were used. A p-value of < 0.05 was set as significant. The statistical analysis was performed using SPSS statistics 16.0 software.

## Results

### Survival, local control and brain control

**Figure 1:**
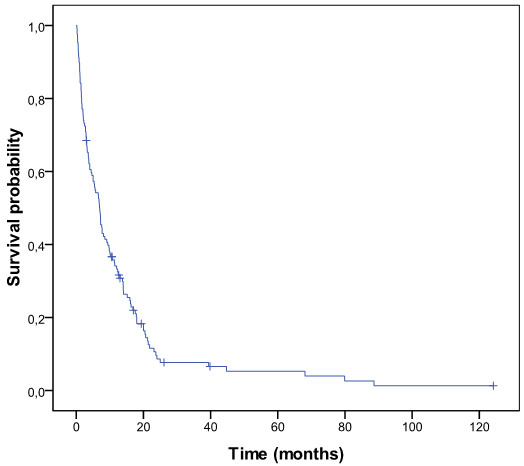
Overall survival: overall survival of 149 patients using the Kaplan-Meier method.

At the end of the follow-up (October 1st, 2009), 117 patients had died, 10 patients were still alive and 22 patients were lost to follow-up. The median survival was 7.0 months (range 0.16-88.6 months). After six months, 53% of the patients were still alive. The 1-year and 5-year survival rates were 33% and 0.4%, respectively. Figure 1 shows the overall survival.

The local control rate in the irradiated volume was 61% after one year. The median survival without local failure was 17.8 months. The control rate in the brain (remote brain control: outside the target volume in the brain, that was treated with stereotactic radiosurgery) was 57% after one year with a median time to any brain failure of 14.0 months.

### Tumor necrosis

In 82 patients (56%), central necrosis was detectable on pretreatment MRI scans. The distribution of patient- and tumor-related parameters and prognostic factors was not different in patients with versus without visible necrosis (Table 3) except the size of the metastatic lesion which was higher in tumors with central necrosis (6.7 cm^3^ vs. 3.2 cm^3^, p = 0.01). Patients with tumor necrosis had a median survival of 5.4 months whereas patients without tumor necrosis had a median survival of 7.2 months. Tumor necrosis (p = 0.001) was a significant prognostic factors for overall survival in the multivariate analysis. This relation still applied if only smaller tumors with a volume below 3.5 cm^3^ were analyzed (p = 0.03). Tumor necrosis had no significant effect on local control (p = 0.3). In a multivariate analysis of all the tumor entities, tumor necrosis is still a significant prognostic factor (p = 0.01) of survival for patients with lung cancer. This prognostic impact could not be detected for the remaining tumor entities.

**Table 3:** Characteristics of patients with or without tumor necrosis

**Parameter**	**N (%) patients without tumor necrosis**	**N (%) patients with tumor necrosis**
Age, years		
Median age	58.4	58.5
Range	23-83	37-81
Status of primary lesion	45 (70)	58 (71)
Active	19 (30)	24 (29)
Controlled		
KPS median	80%	80%
≥ 90	42 (65)	44 (53)
< 90	22 (35)	38 (46)
Number of lesions		
1	43 (67)	58 (70)
2-3	20 (33)	22 (27)
4-6	0	2 (3)
RPA class		
I	6 (9)	11 (13)
II	49 (77)	65 (79)
III	9 (14)	6 (8)
Median dose (Gy)	22.2	22.5
Median tumor size (cm^3^)	3.2	6.7
Treated with WBI	48 (58)	29 (45)
Therapy of primary lesion		
Chemotherapy	65 (79)	49 (76)
Radiotherapy	34 (41)	24 (38)
Operation	52 (63)	42 (65)

Abbreviations:

*KPS* Karnofsky performance status, *RPA* Recursive partitioning analysis, *SRS* stereotactic radiosurgery.

### Side effects

No side effects were noted in 88% of the patients. 11% of the patients had discrete side effects like headache, fatigue or a short-term deterioration of symptoms. In one case, a hemiataxia (NCI-CTC grade 3) appeared as a serious side effect after radiotherapy. A symptomatic improvement was achieved in 37% of the cases, 48% of the patients had persistent symptoms and a symptomatic deterioration was present in 15% of the cases.

### Prognostic factors

Following factors were included in the multivariate analysis: age, KPS, RPA-class, tumor necrosis, additional WBI, the total dose in Gy, the number of brain metastasis and the localization of the lesion in the brain. The Karnofsky performance status (p = 0.001) and the presence of tumor necrosis on pretreatment MRI (p = 0.001) were the most important prognostic factors in the multivariate analysis with the RPA-class and WBI as additional prognostic factors of marginal significance (p = 0.05). Table 4 shows the results of the univariate and multivariate analysis.

**Table 4:** Univariate and multivariate analysis of survival: prognostic factors of survival after SRS

**Factor**	**Univariate**	**Multivariate**
	**p-value**	**p-value**
Age	0.004	0.05
KPS	0.0001	0.001
RPA class	0.001	0.05
WBI	0.04	0.05
Tumor necrosis	0.05	0.001
Localization of lesion	0.007	0.002
Number of brain metastasis	0.02	0.09
Dose	0.002	0.64
Gender	0.46	
Improvement of symptoms	0.45	
Status of primary lesion (active/ controlled)	0.14	
Tumor size	0.47	
Numbers of extracranial metastases	0.43	
Chemotherapy	0.42	
Operation	0.23	
Radiation	0.54	

Abbreviations:

*KPS* Karnofsky performance status, *RPA* Recursive partitioning analysis, *WBI* Whole brain irradiation.

The most important predictor for local control in a multivariate analysis was symptomatic improvement after treatment (p = 0.001). Prognostic factors for brain control outside the irradiated volume were the number of metastases in the brain (p = 0.05), the RPA class (p = 0.05) and the number of extracranial metastases (p = 0.05).

## Discussion

Surgery has been the treatment option of first choice in patients with a single brain metastasis, but radiosurgery has become an equieffective alternative and standard of care for patients with one to three brain metastases [8,24]. Although there are no randomized comparisons to neurosurgical resection, SRS is considered as equally effective [1,18]. The role of whole brain radiotherapy in addition to surgery or SRS remains controversial on the basis of a recent EORTC-study [7,25]. The study, however, suggests that SRS yields at least comparable or even better local control rates than resection, especially if used as single modality without whole brain radiotherapy [7]. Nevertheless, local failures in the treated site occur in about one third of patients. In the EORTC-study, local failure rates after three years were 59% after surgery and 31% after radiosurgery, both without WBI. WBI significantly reduced the failure rates to 27% after resection and 19% after SRS. WBI also significantly reduced failure rate outside the treated volume in the brain and thereby improved brain control, but had no impact on overall survival suggesting that either patients undergoing contemporary diagnostic imaging procedures can effectively be salvaged in case of brain failure or that brain control has a minor impact on survival due to extracranial progression.

The outcome of patients in this series is comparable to data in the literature although they are in general slightly below to recent series. A local control rate of 61% and a brain control rate of 57% are within the range of figures that have been reported in reviews [23]. The overall survival is also within the range of reported results but worse compared to the majority of very recent study [7,15,26]. A likely explanation might be the fact that most of the patients in our series (71%) had an active extracranial disease at the time of radiosurgical treatment, reflecting a possible negative selection bias. Many prospective studies showed that extracerebral control is a significant impact factor of survival [27,28]. Studies, that reported slightly worse results in survival, showed a higher number of patients with an active extracranial disease [29].

Major prognostic factors in patients with brain metastases are the performance status and the activity of extracranial disease with a number of scoring systems [21,22,30]. There are limited data in the literature about the impact of neuroradiological appearance such as MRI morphologic findings and enhancement patterns of the metastatic lesions on outcome. In this analysis, necrosis was defined as non-perfused areas in the tumor on contrast-enhanced MR images. Recent findings in Ewing Tumors support this hypothesis [4]. The authors analyzed the impact of tumor perfusion in MR imaging studies utilizing contrast-enhanced MR images. Necrotic areas were defined as non-perfused regions in the tumor. In Ewing’s sarcomas, the presence of non-perfused (presumably necrotic) areas on pretreatment contrast-enhanced MR images is associated with increased risk of metastases and has prognostic impact in this entity. Nevertheless, it is currently not clear how far macroscopic visualized solid or ring enhancement and microscopic patterns correlate and whether or not they have the same prognostic impact.

In our study, tumor necrosis was a significant prognostic factor of survival. Our study has some limitations because analysis as done retrospectively and the MR examinations were therefore not standardized. Thus, the results should be interpreted with caution, but might serve for hypothesis-generating. Patients with tumor visible necrosis had a median survival of 5.4 months whereas patients without tumor necrosis had a median survival of 7.2 months. A likely explanation might be the effect of hypoxia on radiosensitivity. This hypothesis is supported by findings in other cancer sites. In patients with head and neck tumors undergoing definitive radio- or radiochemotherapy, polarographic measurement of intratumoral pO_2_ is a strong predictor for local control and survival [31]. Moreover, visible necrosis or areas of low density within the gross tumor have prognostic impact [5,32]. In another investigation, the amount of hypoxic volume was the strongest predictor for response to radiotherapy and survival, suggesting that hypoxia impacts on outcome by reducing radiosensitivity [33,34]. These data suggest that visible necrosis within tumors on pretreatment images might be a surrogate marker for relevant radiobiological hypoxia.

Necrosis is the characteristic neuroradiological feature in glioblastoma. A variety of studies has demonstrated a significant impact of necrosis on either progression-free survival or overall survival [6]. However, some investigations have not found an impact of visible necrosis on outcome studies, but this may, at least partly, be explained by the fact that patients with glioblastoma are treated differently with regard to the extent of surgery.

Nevertheless, the situation might be more complex because the prognostic effect of tumor necrosis (as defined here) was - in this analysis - significant only for overall survival and not for local control. The lack of an association between necrosis and local control might be explained by statistical reasons because data on local control were less complete than on survival and an effect on local control might have been missed. On the other hand, the significant effect on survival suggests that the presence of necrosis, besides a possible impact on radiosensitivity, may be an indicator for a more aggressive phenotype. In Ewing tumors treated with radiotherapy, the presence of necrosis on pretreatment MR images was a strong prognostic factor for local control and survival and tumors with necrosis had a significant higher incidence of primary metastases at diagnosis suggesting that the presence of necrosis is associated with a more aggressive behaviour of the disease [4]. However, there was an equal distribution of prognostic factors between patients with or without necrosis in their metastatic lesions in our investigation and this finding does not support the hypothesis that the presence of necrosis is associated with a more aggressive disease. There is currently growing interest in hypoxia imaging with the objective to more precisely deliver radiotherapy to areas with possible radiation resistance and to improve outcome after radiotherapy [35]. If a possible association between visible necrosis and low radiosensitivity could be supported in future investigations, this might add useful information in addition to other imaging modalities for radiotherapy planning.

## Conclusions

In this analysis, the outcome after stereotactic radiosurgery for brain metastases was comparable to data in the literature with a median overall survival of 7.0 months. The presence of tumor necrosis on pretreatment contrast-enhanced MR or CCT images was found to be a prognostic factor for survival irrespective of tumor volume and its impact should be further investigated.

## Abbreviations

CCT: Cerebral computed tomography; CPU: Cancer of unknown primary; KPS: Karnofsky performance status; MRI: Magnetic resonance imaging; NCI-CTC: National cancer institute common toxicity criteria; RPA: Recursive partitioning analysis; SRS: Stereotactic radiosurgery; WBI: Whole brain irradiation

## Competing interests

The authors declare no conflicts of interest.

## Authors’ contributions

KD, KM, JD contribution to design and supervision of the study. KD, KM, TM, DR, VT, MMB, JD contribution to therapy and acquisition of data. KD, KM, JD contribution to acquisition of data and analysis. KD, KM, DP, JD contribution to interpretation of data. KD drafted and reviewed the manuscript and all authors edited and approved the final version.
